# A Smartphone App for Self-Management of Heart Failure in Older African Americans: Feasibility and Usability Study

**DOI:** 10.2196/17142

**Published:** 2020-04-03

**Authors:** Sue P Heiney, Sara B Donevant, Swann Arp Adams, Pearman D Parker, Hongtu Chen, Sue Levkoff

**Affiliations:** 1 College of Nursing University of South Carolina Columbia, SC United States; 2 College of Nursing University of Arkansas for Medical Sciences Little Rock, AR United States; 3 Environment and Health Group Boston, MA United States; 4 College of Social Work University of South Carolina Columbia, SC United States

**Keywords:** heart failure, mobile health app, self-management

## Abstract

**Background:**

Mobile health (mHealth) apps are dramatically changing how patients and providers manage and monitor chronic health conditions, especially in the area of self-monitoring. African Americans have higher mortality rates from heart failure than other racial groups in the United States. Therefore, self-management of heart failure may improve health outcomes for African American patients.

**Objective:**

The aim of the present study was to determine the feasibility of using an mHealth app, and explore the outcomes of quality of life, including self-care maintenance, management, and confidence, among African American patients managing their condition after discharge with a diagnosis of heart failure.

**Methods:**

Prior to development of the app, we conducted qualitative interviews with 7 African American patients diagnosed with heart failure, 3 African American patients diagnosed with cardiovascular disease, and 6 health care providers (cardiologists, nurse practitioners, and a geriatrician) who worked with heart failure patients. In addition, we asked 6 hospital chaplains to provide positive spiritual messages for the patients, since spirituality is an important coping method for many African Americans. These formative data were then used for creating a prototype of the app, named Healthy Heart. Specifically, the Healthy Heart app incorporated the following evidence-based features to promote self-management: one-way messages, journaling (ie, weight and symptoms), graphical display of data, and customized feedback (ie, clinical decision support) based on daily or weekly weight. The educational messages about heart failure self-management were derived from the teaching materials provided to the patients diagnosed with heart failure, and included information on diet, sleep, stress, and medication adherence. The information was condensed and simplified to be appropriate for text messages and to meet health literacy standards. Other messages were derived from interviews conducted during the formative stage of app development, including interviews with African American chaplains. Usability testing was conducted over a series of meetings between nurses, social workers, and computer engineers. A pilot one-group pretest-posttest design was employed with participants using the mHealth app for 4 weeks. Descriptive statistics were computed for each of the demographic variables, overall and subscales for Health Related Quality of Life Scale 14 (HQOL14) and subscales for the Self-Care of Heart Failure Index (SCHFI) Version 6 using frequencies for categorical measures and means with standard deviations for continuous measures. Baseline and postintervention comparisons were computed using the Fisher exact test for overall health and paired t tests for HQOL14 and SCHFI questionnaire subscales.

**Results:**

A total of 12 African American participants (7 men, 5 women; aged 51-69 years) diagnosed with heart failure were recruited for the study. There was no significant increase in quality of life (*P*=.15), but clinically relevant changes in self-care maintenance, management, and confidence were observed.

**Conclusions:**

An mHealth app to assist with the self-management of heart failure is feasible in patients with low literacy, low health literacy, and limited smartphone experience. Based on the clinically relevant changes observed in this feasibility study of the Healthy Heart app, further research should explore effectiveness in this vulnerable population.

## Introduction

### Background

Self-monitoring, generally defined as the awareness of symptoms through measurements, recordings, and observations, is foundational to the successful management of any chronic health condition. Mobile health (mHealth) apps are dramatically changing how patients and providers manage and monitor chronic health conditions, especially in the area of self-monitoring [1]. In particular, mHealth apps provide patients with quick and easy access to real-time health information to assist in making health decisions (ie, when to call the physician or go to the emergency department) and improve patient outcomes. Recent studies suggests that use of mHealth apps is associated with reductions in mortality and hospitalizations, along with improved adherence to therapy and enhanced quality of life [2,3].

### African American Patients With Heart Failure: A Vulnerable Population

Adults with heart failure represent a particularly vulnerable population that may benefit from mHealth apps, especially African American adults living in rural communities. Currently, heart failure is the leading cause of hospitalization for older adults, which can lead to an increased risk of complications and health care costs [4,5]. The highest rates of hospitalization for patients with heart failure are reported in the southeast of the United States, including South Carolina [6]. In addition to high poverty levels, African American patients with heart failure may experience other challenges that negatively impact their health status, including lack of insurance, limited or no transportation, and an inadequate supply of health care providers [7]. Conversely, overall health could be improved if health care providers harness the cultural basis of self-care in the African American population, such as spirituality, social support, and nonbiomedical healing traditions [8].

The use of mHealth apps has successfully assisted in the self-management of patients with heart failure [9,10]. However, there is limited literature to support the use of mHealth in the African American population [11-14], with few heart failure mHealth apps designed specifically for this population, who are typically sicker, poorer, less educated, and more affected by comorbid conditions compared to other racial groups in the United States [15]. Therefore, there is a need for culturally appropriate mHealth apps to assist this vulnerable population of African American patients with heart failure.

The purpose of this study was to design and describe the feasibility of the Healthy Heart app in African American patients with a discharge diagnosis of heart failure and assess impacts of using the app on quality of life and self-care.

### App Development

To overcome these concerns and limitations, our team developed a prototype app, termed Healthy Heart, through an iterative design process with input from end users (ie, patients with heart failure and health care providers). The team consisted of nurses, social workers, and computer engineers. We adopted the situation-specific theory for behavioral change, which emphasizes the following three key components of self-management: (1) self-monitoring of symptoms, which helps patient to notice and focus on specific health problems; (2) health literacy, which educates patients about the role of healthy behaviors leading to desirable health benefits; and (3) confidence (self-efficacy), which is associated with making behavioral changes and taking actions to manage and improve one’s health [16]. App development was also guided by emerging mHealth research to deliver interventions for self-management by fostering the cognitive and behavioral changes necessary to promote stability, health, and well-being in patients with heart failure [17,18].

**Figure 1:**
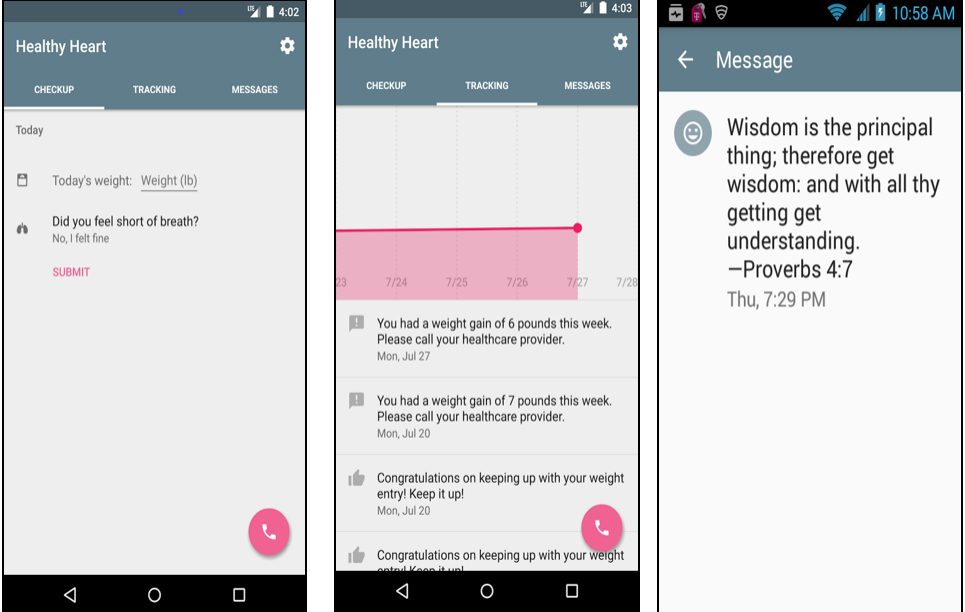
Screenshots of sample app features.

Prior to app development, we used purposeful sampling to recruit 7 African American patients with heart failure and 3 African American patients with cardiovascular disease for qualitative interviews about their experience with heart failure. We also recruited 6 health care providers (cardiologists, nurse practitioners, and a geriatrician) who worked with heart failure patients. Using these formative data, we created a prototype of the Healthy Heart app incorporating the following evidence-based features to promote self-management: one-way messages, journaling (ie, weight and symptoms), graphical display of data, and customized feedback (ie, clinical decision support) based on daily or weekly weight. Figure 1 displays screenshots of these main features.

The educational messages about heart failure self-management were derived from the heart failure teaching material provided to patients diagnosed with heart failure, and included information on diet, sleep, stress, and medication adherence. Sources for patient teaching included Agency for Healthcare Research and Quality Heart Failure Communication Tools [19], Health Topics, Heart Failure [20], and How to Reduce Sodium [21]. The information was condensed and simplified to be appropriate for text messages and to meet health literacy standards. Other messages were derived from interviews conducted during the formative stage of app development, including interviews with 6 African American hospital chaplains. We involved chaplains at this stage because spirituality is an important component of African American culture. For example, one motivational message was “Even when we go through things God is always there to lead us through troubling times in our lives” paraphrased from Psalms 46. The team conducted usability testing over a series of meetings between the nurses, social worker, and computer engineers.

## Methods

### Overview of Study Design

In this quasiexperimental pilot study using a one-group pretest-posttest design, 12 African American participants with a discharge diagnosis of heart failure used the Healthy Heart app. Prior to start of the study, Internal Review Board approval was received from the University of South Carolina and a locally owned, not-for-profit health care system.

### Recruitment and Retention

Inclusion criteria were English-speaking African American men or women aged 50 years or older who received a diagnosis of heart failure and were discharged from the hospital to home care. Patients were excluded if they had a major cognitive impairment or a current diagnosis of psychosis. The home health nurse did not use a screening tool to determine cognitive impairment but instead used the discharge summary and her clinical judgment with the participant and family. Subjects who could not speak either English or a dialect of English were also excluded. Patients younger than 50 years of age were excluded from the study with no restriction on the upper age for participants.

The pool of potential participants comprised patients diagnosed with heart failure and discharged from the locally owned, not-for-profit health care system receiving 30 days of home health telemonitoring (ie, standard of care). Home health telemonitoring included an interactive monitor with direct connections to a scale and blood pressure cuff that automatically uploaded the data to a home health nurse. However, no additional visits were made to the patients after the monitor was installed and set up. The patients received telephone communication with the nurse if any of the measures were abnormal.

The home health nurses collaborated with the study team by querying the telemonitored patients of interest in participating in the Healthy Heart app study. Based on collaboration with the home health facility, we were unable to collect data about the number of patients approached. Recruitment occurred between November 2015 and September 2016. If interested, the patient signed a Health Information Portability and Accountability Act release, which included contact information for the study coordinator, and received a fact sheet about the study. The study coordinator called the participants for a phone discussion that summarized and explained the study details, including the purpose, procedures, risks, benefits, and voluntary participation. Potential participants were encouraged to ask any questions. If the participant was interested, an appointment was scheduled for the study coordinator to visit the participant’s home to obtain consent and administer the baseline study assessments. All participants were provided with an Android phone with the Healthy Heart app (ie, intervention) installed. This provided consistency and ease of instructing the participants on using the app. No monetary compensation was offered to the study participants; however, they were able to keep the low-cost study phone after the study period. In addition, a small thank you postcard and gift (approximately $1 value) was mailed to the participants weekly to promote retention in the study.

### Procedures

Once the study coordinator received verbal consent from the participant during the telephone conversations, the first home visit was scheduled within approximately 2 weeks after hospital discharge while the patient was still receiving telemonitoring from the home care nurse. During this home visit, the study coordinator obtained all baseline data.

A second home visit occurred approximately 1 week later in which the participant received the prepaid activated phone. The coordinator educated the participant about using the phone and Healthy Heart app. The study coordinator spent time teaching the participant (and caregiver if desired) how to turn the phone on and off, how to charge the battery, how to open the Healthy Heart app, and so forth. The US Department of Health and Human Services reports nearly 9 out of 10 adults in the United States have difficulty using everyday health information, and South Carolina ranks 39th in literacy in the country [22]. Based on our previous work with low-literacy, low-health literacy, and low-digital literacy patients, we anticipated that these participants would have low familiarity with using a smartphone or an app. Indeed, 7 of the 12 participants did not previously own a smartphone. Thus, we designed an instruction booklet written at a sixth-grade or lower reading level and used screenshots to explain each operation of the phone and each function of the app. In addition, the study coordinator assisted the participants with entering the phone numbers of the health care provider and pharmacy. On average, these visits lasted 1-2 hours. This home visit was timed so that the participant would have 1 week of overlap with the home health telemonitoring (ie, standard of care) and start of self-monitoring via the Healthy Heart app (ie, intervention). This overlap allowed the participants to become familiar with the app before stopping the telemonitoring for a seamless transition. The participant was instructed to call the coordinator if any questions arose. The coordinator called the participants about 2 weeks after the initial training to ascertain if they were experiencing any difficulties using the phone and the app. No additional visits for training occurred, but the coordinator was available during normal business hours via phone to assist with any usability or technical issues.

Six weeks after the initial assessment, a final assessment was completed, including the completion of assessment instruments obtained at baseline, the participants’ perceptions of the phone and Healthy Heart app, and retrieval of data.

### Description of the Intervention

The intervention consisted of the use of all app features continuously over 4 weeks. A total of 3 messages were sent daily: (1) a reminder to weigh every morning, (2) an educational message, and (3) motivational messages. Messages were repeated when the message bank had sent all messages once. The other two components of the app were journaling and customized feedback. Journaling consisted of the participant entering a daily weight and responding to a question about shortness of breath. The final component was customized feedback (ie, clinical decision support) that sent an alert message if the weight exceeded standards set by the home health protocol for monitoring heart failure. The message instructed the participant to call the health care provider if they experienced an increase of 2-3 pounds in a day or over 5 pounds in a week. Regardless of the amount of weight gained or lost, the participant could view a weekly graph of weight fluctuations.

### Data Collection

Demographic and personal data were collected using a demographic data form adapted from a previous study [23]. Examples of personal data include living situation, instrumental social support, years since diagnosis, phone type, insurance type, and faith group. In addition, the participants were interviewed to ascertain their current recall of prior teaching on heart failure and current information on how to handle heart failure-related concerns. The interview consisted of yes/no and open-ended questions (eg, “Has anyone talked with you about the things you need to know in order to take care of yourself and your heart?”).

The 14-item Centers for Disease Control and Prevention (CDC) Health Related Quality of Life Scale 14 (HRQOL14) was used to assess the participants’ quality of life [24]. This scale has strong validity and reliability [25]. The following three modules of the HRQOL14 were utilized: core healthy days, activity limitations, and healthy days symptoms. The core healthy days (4 items) assessed (1) perceived general health, (2) number of days when physical health was perceived as poor, (3) number of days when mental health was perceived as poor, and (4) number of days when the respondent perceived activity limitations due to poor mental or physical health. Instructions for scoring followed the CDC guidelines [24]. The activity limitations module assessed more details about activity limitations due to poor health, including personal care and routine needs. The healthy days symptoms module was assessed only if the participant responded “no” to the activity limitation item in the healthy days module. For both the activity limitations and healthy days symptoms modules, we used the CDC Statistical Analysis System syntax for scoring [26].

The Self-Care of Heart Failure Index (SCHFI) Version 6 [16,27-29] subjectively assesses the participant in three areas: maintenance, management, and self-confidence, which are based on Riegel and Dickson’s [16] conceptual model of heart failure self-care. The instrument consists of 22 items rated on a 4-point scale with each subscale scored separately. Barbaranelli et al [27] recommended against reporting an overall score when a participant does not have a certain number of problems. The raw score ranges for each subscale are as follows: 10-40 for self-care maintenance, 4-24 for management, and 6-26 for self-care confidence. These ranges were all standardized to 0-100. Instructions for scoring and methods for handling missing data followed the method of Riegel and colleagues [28]. Reliability of the subscales ranged from low to adequate (α=0.56-0.80) and construct validity was adequate [29].

### Analysis

Descriptive statistics were computed for each of the demographic variables, overall and subscales for HQOL14, and subscales for the SCHFI using frequencies for categorical variables and means (SD) for continuous variables. Baseline and postintervention comparisons were computed using the Fisher exact test for overall health and with paired *t* tests for HQOL14 and SCHFI subscales.

## Results

### Recruitment

We enrolled 12 African American participants for this feasibility study. One participant was lost to follow up at the second assessment. Thus, 11/12 (92%) participants completed the study.

### Demographic and Health Characteristics of the Sample

The main characteristics of our sample are summarized in Table 1. There were more men than women, and the age of participants ranged from 51 to 69 years. One quarter of the participants were married and the remaining participants were single, divorced, separated, or widowed. The majority of participants (10/12, 83%) graduated from high school. Over half of the participants (8/12, 67%) preferred not to answer about income or did not know their income. One third of the participants were on Medicare or Medicaid (4/12), one third had no insurance (4/12), one fourth had private insurance, and one third (4/12) did not know about insurance coverage. All participants (12/12, 100%) reported belonging to a church. Eleven of the 12 participants (92%) owned a cell phone, and 5/12 (42%) owned a smartphone. The majority of the participants received a diagnosis of heart failure less than one year prior to the study (7/12, 58%).

**Table 1:** Demographic and health characteristics of the study population (N=12).

Variable		Value
Age (years), mean (SD)		57.83 (1.68)
**Gender, n (%)**		
	Male	7 (58)
	Female	5 (42)
**Marital status, n (%)**		
	Married	3 (25)
	Not married	9 (75)
**Education, n (%)**		
	Less than high school	2 (17)
	High school	7 (58)
	More than high school	3 (25)
**Income, n (%)**		
	Do not know	2 (17)
	Less than $10,000	3 (25)
	$30,000-$39,999	1 (8)
	Chose not to answer	6 (50)
**Years since diagnosis, n (%)**		
	<1	7 (58)
	2 to 8	3 (25)
	11 to 13	2 (17)

### Heart Failure Care Activities

In the baseline assessment, most participants (11/12, 92%) stated that their health care provider (ie, doctor, nurse, or dietician) had discussed ways to manage their heart failure. All participants reported weighing themselves daily, but 3/12 (25%) noted that they did not record their weight regularly. Six participants (50%) received instruction on specific limits for fluid intake and provided examples of 40 ounces, a quart, a gallon, or a liter. However, only 5/12 (42%) participants reported that they followed the recommended fluid restriction. Participants gave a variety of answers when asked about salt restriction (eg, restricted salt altogether, do not eat much salt, or restricted to 200 mg per day).

### Feasibility of App Usage

Participants with low digital literacy (ie, nonadopters of digital technology) initially struggled with phone and app operation. Anticipating this prior to phone distribution, we removed all nonessential apps from the phone to alleviate confusion about which app to use: the distributed phone only included the ability to call, a search engine app, and the Healthy Heart app. Nevertheless, participants had to practice several times during the training to understand how to answer the phone, make a phone call, and open the app. They also needed to use the instruction booklet to help remember these steps in operating the phone. In spite of these barriers, over 60% of the data were successfully collected.

At the end of the intervention, we evaluated the participants’ use of the phone and app to assist with future iterations of the app as well as to guide future research in working with this population. Unfortunately, we were unable to identify an evaluation tool specifically for mHealth apps and this population. For example, the Systems Usability Scale [30,31] was too broad to help us identify specific problems with the phone and the app. We asked closed questions that assessed potential problems with the phone and messages (ie, readability), and these results are summarized in Table 2.

**Table 2:** Postintervention experience with the project and phone usage (N=11).

Question	Response	n (%)
Did you have any difficulty using the phone?	No	6 (55)
Was it easy to charge the phone?	Yes	10 (91)
Was it easy to read the message?	Yes	11 (100)
Was the font (size of the print) big enough?	Yes	10 (91)
Was the length of the message too long, not long enough, just right?	Just right	10 (91)
Have you used the phone to go online (search the web)?	Yes	4 (36)
Have you made any phone calls with this phone?	Yes	10 (91)
Have you sent any text messages?	Yes	4 (36)
Have you seen the Quick Dial feature (ie, that you can press to call your health providers)?	Yes	5 (46)
If yes, have you ever used the Quick Dial feature?	Yes	2 (18)
If not, would you use it if you need to contact your providers?	Yes	6 (55)

### Quality of Life and Self-Care Heart Failure Index

For HRQOL14, at baseline, 5/12 (42%) of participants reported that their overall health was poor or fair, whereas at the postintervention assessment, 2/11 (18%) rated their health as poor or fair. No significant changes were found for any of the HQOL14 subscales. At baseline, participants reported a mean of 4 (SD 5.75, range 0-14) physically unhealthy days. Postintervention, the mean number of unhealthy days was reduced to 2.54 (SD 4.32, range 0-15). At baseline, respondents reported a mean of 1.42 mentally unhealthy days (SD 2.39, range 0-7), which increased postintervention to 3.18 (SD 9.02, range 0-30). At baseline, the mean number of days participants reported experiencing limitation in daily activities due to poor physical or mental health was 2.58 (SD 3.03, range 0-8), which decreased postintervention to 1.73 (SD 3.00, range 0-10). The mean preintervention combined physical and mental unhealthy days was 5.41 (SD 7.54, range 0-21), which was similar to that postintervention at 5.54 (SD 9.95, range 0-30).

For the SCHFI, we were not able to analyze the management subscale due to participant responses. Per Riegel et al [28], if a participant responded “no” to experiencing trouble breathing or ankle swelling in the past month, then the management section of the SCHFI was not scored. Only 5/12 (42%) participants reported problems with one of these symptoms. Therefore, we compared baseline and postintervention scores for maintenance and confidence. No significant differences were found on either subscale. However, based on Riegel et al [28], the maintenance, management, and self-confidence scales showed clinically relevant improvement from baseline to posttest as the difference in the mean baseline score for maintenance and management was greater than 8 (Table 3).

**Table 3:** Analysis of subscales of Self-Care of Heart Failure Index.

Subscale	Baseline, mean (SD)	Postintervention, mean (SD)	*P* value	Difference score	Clinically relevant^a^
Maintenance (N=12)	63.33 (15.50)	72.70 (15.62)	.15	9.37	Yes
Management (N=9)	70.0 (7.07)	85.00 (14.36)	Presample too small^b^	15.00	Yes
Confidence (N=12)	83.86 (13.51)	90.90 (10.90)	.17	7.04	Approaches

^a^Clinical relevance defined as at least an 8-point difference in baseline to postintervention.

^b^At least half the items were missing from participant responses.

### Hospital Readmission and Emergency Room Visits

During the final assessment, we ascertained information about emergency room and hospital readmissions. One participant reported admission to the hospital via the emergency room and another other participant reported an emergency room visit during the 6 weeks of the study. Six participants called their provider at least once based on instructions from the app.

## Discussion

### Feasibility

When working with a digitally naïve population with low literacy, we recommend careful consideration of the phone display (ie, simple and uncluttered) and the use of simple instructional materials. The use of screenshots instead of text in the instructional materials seemed to help participants understand how to use the phone and app. Although such work may be challenging, more research is needed on designing mHealth for this population. Our study makes a significant contribution toward the use of mHealth apps for managing a chronic disease in a health-disparate population. In this regard, we substantiated the work by Cajita et al [32] showing that older adults are willing to use mHealth technology.

### Exploratory Findings

The Healthy Heart app did not significantly increase quality of life (*P*=.15), but did show clinically relevant changes in heart failure self-care maintenance, management, and confidence. Our preliminary findings are similar to those of Dang et al [33] who identified that an mHealth app is a feasible method of assisting health-disparate patients to manage their disease. However, in contrast to the results of their randomized controlled trial [34], our study participants showed clinically relevant changes in their ability to maintain and respond to heart failure symptoms, but did not show significant improvements in self-efficacy or quality of life.

The participants in our study had low rates of hospital readmissions and emergency room visits. This finding is consistent with a systematic review showing that that mHealth apps to manage heart failure may reduce heart failure-related hospital days [35]. However, our baseline findings regarding participant understanding are inconsistent with those of Spaling et al [36] who found that patients could recall advice about self-care. Our participants’ recall was not clear and often contained vague information. Our participants showed similar improvement in self-care to that found in the Spaling et al [36] study, although our change was not statistically significant.

### Limitations

One limitation of the study is that we only tested the intervention (use of the app) for 4 weeks. This may not have allowed enough time for behavioral changes to be established and influence quality of life. Other limitations include the small sample size and the quasiexperimental nature of the study. However, previous studies suggest that a quasiexperimental design is appropriate when participants are unlikely to change their behavior without an intervention (ie, inclusion of a usual care group would not add information during pilot testing) [37,38]. Further, the impact of prior experience with telemonitoring (ie, standard of care) could have influenced the outcomes of this study. However, since we collected baseline data at the end of telemonitoring, we feel confident that our data reflected important improvements related to the use of the Healthy Heart app.

### Conclusions

This study provides insights into the feasibility of mHealth apps among a disparate population with low literacy, low health literacy, and limited smartphone use. Additionally, the study provides essential lessons for the use of mHealth apps in this population. Providing instructions with screenshots with minimal text was a successful technique for training the participants on using the smartphone and Healthy Heart app. Furthermore, the inclusion of culturally appropriate messages potentially enhanced the acceptance of the Healthy Heart app. The initial focus groups with African American patients guided the development of the mHealth app to include culturally appropriate content. These findings suggest that patients want content that recognizes cultural differences. This study demonstrated clinically relevant changes in heart failure self-care maintenance, management, and confidence. As mHealth technology continues to advance, it is important to take into account how to address the needs of all patients, including those with low literacy, low health literacy, and limited smartphone use, as well as cultural differences. This study can provide some guidance on addressing these challenges for unique patient populations.

## Abbreviations

CDC: Centers for Disease Control and Prevention
HRQL14: Health Related Quality of Life Scale 14
mHealth: mobile health
SCHFI: Self-Care of Heart Failure Index
